# Current Levels of Environmental Exposure to Cadmium in Industrialized Countries as a Risk Factor for Kidney Damage in the General Population: A Comprehensive Review of Available Data

**DOI:** 10.3390/ijms24098413

**Published:** 2023-05-08

**Authors:** Nazar M. Smereczański, Małgorzata M. Brzóska

**Affiliations:** Department of Toxicology, Medical University of Bialystok, Adama Mickiewicza 2C Street, 15-222 Bialystok, Poland

**Keywords:** cadmium, kidney, nephrotoxicity, biomarkers of nephrotoxicity, chronic kidney disease, environmental exposure, general population, LOAEL, NOAEL, odds risk

## Abstract

The growing number of reports indicating unfavorable outcomes for human health upon environmental exposure to cadmium (Cd) have focused attention on the threat to the general population posed by this heavy metal. The kidney is a target organ during chronic Cd intoxication. The aim of this article was to critically review the available literature on the impact of the current levels of environmental exposure to this xenobiotic in industrialized countries on the kidney, and to evaluate the associated risk of organ damage, including chronic kidney disease (CKD). Based on a comprehensive review of the available data, we recognized that the observed adverse effect levels (NOAELs) of Cd concentration in the blood and urine for clinically relevant kidney damage (glomerular dysfunction) are 0.18 μg/L and 0.27 μg/g creatinine, respectively, whereas the lowest observed adverse effect levels (LOAELs) are >0.18 μg/L and >0.27 μg/g creatinine, respectively, which are within the lower range of concentrations noted in inhabitants of industrialized countries. In conclusion, the current levels of environmental exposure to Cd may increase the risk of clinically relevant kidney damage, resulting in, or at least contributing to, the development of CKD.

## 1. Introduction

Epidemiological studies have provided a growing amount of reliable evidence that long-term moderate and, sometimes, even low-level environmental exposure to certain chemicals poses a risk to health and can result in damage to various organs and systems, including the kidneys [1,2,3]. Therefore, an important task in the area of public health is to estimate the threat posed by chemical substances present in the living and work environments of humans, as well as in food, in economically developed and developing countries. The proper assessment of the health risks posed by these pollutants is necessary in order to find and implement appropriate preventive strategies and effective methods for treating their unfavorable effects. Recognizing the risk factors for kidney damage in the general population is also important because of the possibility of simultaneous exposure to two or more nephrotoxic factors that could interact, leading to an intensification of the injurious impact on this organ [4,5,6,7].

Among the xenobiotics to which the general population is exposed throughout their lifetimes and that can be harmful to the kidney, special attention has been paid to toxic heavy metals, including cadmium (Cd). Cd was ranked seventh on the 2022 priority list of hazardous substances of the Agency for Toxic Substances and Disease Registry [8], and forecasts indicate that the exposure of the general population to this element is increasing in industrialized and developing countries [9,10]. The results of epidemiological studies, especially recent findings, show that even low-level chronic exposure to this xenobiotic can result in damage to the kidney, liver, skeletal system, cardiovascular system, and nervous system, as well as deterioration in hearing and sight (for a review, see [2,11,12,13,14,15]). Moreover, exposure to this element has been suggested to contribute to the development of cancer [16].

The kidney is the main location of Cd accumulation in the body, as well as the target organ (i.e., the organ damaged first) for this element during chronic intoxication [15,17,18,19]. The fact that repeated high or moderate exposure to this xenobiotic injures this organ is well known [20,21,22,23,24,25]. However, for an assessment of the health hazard faced by the general population, clarifying whether and to what extent the low-level lifetime exposure to Cd that is currently common and unavoidable in industrialized countries [11,13,26,27] could increase the risk of damage to various organs and systems, especially the kidney, is particularly important. This is also important because evidence suggests that the level of exposure to this metal that is currently recognized as safe may be too high and should be revised [2,28,29,30].

Due to its role in the elimination of exo- and endogenous substances and products of their biotransformation from organisms, the kidney is an organ whose proper function determines the general state of health [31,32,33]. Chronic kidney disease (CKD), also called chronic kidney failure, is a global problem, as it is a leading cause of death in both developed and developing countries [34,35]. Therefore, considering the increasing prevalence of kidney diseases in the world’s population [34,35,36], we must identify all causative factors, including Cd, which deserves special attention among the potential risk factors for kidney dysfunction [20,22,31,37]. In the case of xenobiotics, estimating the exposure levels at which this effect occurs is also crucial.

Thus, the aim of this paper was to provide a critical review of the literature available worldwide on the influence of current human exposure to Cd in economically developed and developing countries on the kidneys. In addition, we aimed to assess, based on reliable data, whether this exposure poses a substantial risk of clinically relevant damage to this organ. For this purpose, ample evidence from recent epidemiological studies on this topic is presented and discussed. Since the adequate assessment of the impact of Cd on the kidney and the risk of injury to this organ at low exposure levels requires the measurement of early and sensitive biomarkers, special attention was also paid to providing an overview of the available data on biomarkers of Cd nephrotoxicity in terms of their usefulness in detecting early changes under low exposure levels. We attempted to select the earliest and most useful of these biomarkers. A critical review of the methods of estimating the intensity of exposure to this element, including biomarkers of exposure, is also provided. From a public health perspective, evaluating the concentration of Cd in the urine and blood of the general population that poses a substantial risk of damage to the kidney is crucial. Hence, we assessed, based on the available epidemiological data, the no observed adverse effect level (NOAEL) and lowest observed adverse effect level (LOAEL) of the Cd concentration in the blood and urine for clinically relevant kidney damage. This article is the first review focused on evaluating the risk of kidney damage at low-to-moderate levels of environmental exposure to Cd, which occur in numerous countries worldwide.

The following literature databases were searched to prepare this review article: Pubmed, Scopus, Elsevier, and Taylor & Francis Online. We used keywords such as cadmium and kidney, nephrotoxicity, general population, environmental exposure, occupational exposure, concentration, blood, urine, health risk, health effects, accumulation, target organ, NOAEL, LOAEL, threshold level, mechanisms of action, damage, injury, dysfunction, disease, proximal tubules, glomerulus, nephron, markers of nephrotoxicity, oxidative stress, apoptosis, metallothionein (MT), β2-microglobulin (β2-MG), retinol-binding protein (RBP), α1-microglobulin (α1-MG), N-acetyl-β-D-glucosaminidase (NAG), kidney injury molecule-1 (KIM-1), glomerular filtration rate (GFR), estimated glomerular filtration rate (eGFR), and albuminuria. The assessment of whether the current levels of environmental exposure to Cd in industrialized countries may be a risk factor for kidney damage, including CKD, was based on an overview of data published within the last 10 years (the search strategy is presented in Table 1). Reports from studies conducted among inhabitants of highly polluted areas, presenting high concentrations of Cd in the blood and urine, were excluded from this review because this exposure level was unrepresentative of the general population worldwide.

## 2. The Kidney as One of the Most Important Organs in the Body

**Figure 1:**
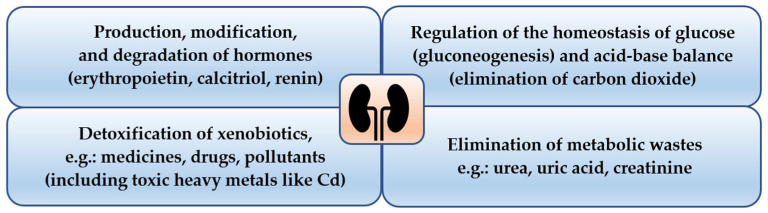
Functions of the kidney.

The kidney is one of the most important organs in the body, playing a multidirectional role (Figure 1) [31,38]. The function of the kidney in the process of detoxification mainly consists of the elimination of toxic substances and their metabolites, as well as the biotransformation of xenobiotics [33,39]. Although the liver is the main organ responsible for the biotransformation of xenobiotics in the body, the kidney is also involved in this process. Moreover, the kidney is the location for the accumulation of numerous substances, including toxic heavy metals such as Cd [17,19,40,41,42]. The accumulation of toxic substances in the kidneys is, to some extent, a process of detoxification (e.g., Cd retention in the MT-bound form), because the substances accumulated in this organ are excluded from systemic circulation; however, the ability of the kidneys to accumulate xenobiotics is limited, and this process generally has negative outcomes for this organ [27,40]. Furthermore, substances accumulated in the kidneys can be released into the bloodstream and exert a toxic effect [43].

The kidney is at a particularly high risk of being damaged by xenobiotics because of the crucial role of this organ in the detoxification of chemical substances and the elimination of unnecessary compounds from the body [31,32,44]. On the one hand, exposure to xenobiotics can damage the kidney. On the other hand, the functional state of this organ determines the unfavorable effects of the toxic substances to which the body is exposed, as well as their metabolites [20,45]. Regardless of the cause, the dysfunction of the kidney leads, or at least contributes, to the development of anemia, cardiovascular diseases, and diabetes, and damages mineral and bone metabolism [46]. Additionally, kidney failure enhances the toxicity of chemical substances, mainly by prolonging their halftime in the body through slower excretion [31,47].

## 3. Main Causes of Kidney Dysfunction in the General Population

Kidney dysfunction (kidney failure) is a gradual loss in the functionality of this organ due to endo- or exogenous causes. This condition may include morphological and functional changes, and may be acute or chronic [38,47,48]. CKD is a long-term condition in which the kidneys are damaged and cannot filter blood as they should. It is diagnosed based on albuminuria and a decreased renal filtration rate (GFR or eGFR). CKD is a major worldwide health problem, with a prevalence of 11–13% globally, of which only 10% are diagnosed and receive proper treatment [36,49].

The destructive impact of various factors on the kidney involves changes in the tubules and glomeruli of the nephrons, such as modifications to the glomerular hemodynamics, oxidative injury to tubular and glomerular cells, thrombotic microangiopathy, and rhabdomyolysis [9,33,44]. Among the main causes of kidney dysfunction in the general population (Table S1), diabetes [50] and hypertension [51] are considered the most common; however, medicines and environmental or occupational pollutants, including Cd [32,33,48], should not be uncredited. Moreover, co-exposure to multiple nephrotoxic factors increases the risk of kidney damage [4,6], and Cd may be one of these factors [5,52], as explained later in this review.

## 4. The Current Cd Exposure Level in Industrialized Countries

The technological progress in recent decades is the main reason for the increased use of Cd worldwide, contaminating the environment and dietary products and resulting in inevitable lifelong human exposure to this xenobiotic [2,9,26,53,54]. Naturally, Cd is present in the lithosphere at low concentrations (0.15 mg/kg in the Earth’s crust and 1.1 × 10^−4^ mg/L in seawater) [9], but numerous industrial activities (e.g., mining and smelting) have increased its presence in the environment and enhanced human exposure [9]. Every year, thousands of tons of Cd-contaminated wastes are discarded into the environment worldwide [9,21]. Despite the actions taken to remove Cd from and decrease the amount of Cd released into the lithosphere, the contamination of the natural environment with this xenobiotic shows an increasing trend, as this metal is not biodegradable and persists in the environment for hundreds of years [55].

Foods, especially plant products, are the main source of exposure to this heavy metal in the non-smoking portion of the general population [2,9,12], while for habitual tobacco smokers, tobacco smoke is a serious additional and often main source of intoxication with this xenobiotic [56,57]. The available data indicate that the current dietary intake of Cd worldwide sometimes exceeds the levels acknowledged to be safe [11,58,59]. The provisional tolerable monthly intake (PTMI) for this heavy metal is 25 μg/kg body weight (b.w.) [59], while its provisional tolerable weekly intake (PTWI) according to the European Food Safety Authority (EFSA) is 2.5 μg/kg b.w. [25]. Currently, the dietary intake of Cd in populations inhabiting areas considered to be non-polluted varies from 10 to 70 μg/day [25,26,29,54,60,61,62,63]. Assuming an average body weight of 70 kg, the weekly and monthly intake of this heavy metal would reach 1–7 and 4.2–30 μg/kg b.w., respectively. This proves that even for inhabitants of areas that are not polluted with Cd, the safe intake levels of this toxic element (the PTWI and PTMI) may be exceeded, in some cases by about threefold (PTWI). The lowest daily intake of Cd, with an arithmetic mean (AM) oscillating around 10 μg (1 μg/kg b.w./week; 99th percentile—2.1 μg/kg b.w./week), was noted in Sweden [61]. The highest oral exposure to this xenobiotic (exceeding the PTMI for this element by more than two-fold), which reached 55 μg/kg b.w./month in males and 53 μg/kg b.w./month in females (aged 18–39 years), was noted in industrialized regions of China [59]. The facts that the Cd concentration in commercially available dietary products sometimes exceeds the safe-limit values and that the dietary intake of this xenobiotic in some parts of the world or in certain groups exceeds the levels currently recognized as safe (the PTWI and PTMI) indicate a substantial risk of excessive intoxication with this element [54,59,62,64].

Numerous factors may increase the gastrointestinal absorption of Cd, simultaneously enhancing the burden of this xenobiotic in the body and exacerbating the risk of toxic effects. The efficiency of the absorption of this xenobiotic from the gastrointestinal tract is low, reaching only 1 to 8% in humans, and it depends mainly on diet, age, and sex [26,27,65,66,67,68]. Enhanced Cd absorption is noted particularly in women of reproductive age and children [67,68]. Among the nutritional factors influencing the gastrointestinal absorption of this heavy metal, the presence of essential elements (mainly zinc, magnesium, selenium, calcium, and iron); vitamins; and other bioactive compounds, such as polyphenols, phytates, and carotenoids, is the most important (for a review, see [69,70]). The bioavailability of Cd from the digestive tract may be increased by up to 20% due to the insufficient consumption of these nutritional factors [65,66].

Habitual tobacco smoking significantly increases the burden of Cd in the body, as each cigarette contains approximately 1 μg of this element, 25–35% of which undergoes absorption into the bloodstream [47]. Substantial data show that tobacco smoking is a source of exposure to large quantities of Cd. Concentrations of this heavy metal found in the blood and urine of active smokers were two to eight times higher compared to non-smokers who were gender- and ethnicity-matched and/or living in the same area (Table 2). Exposure to second-hand cigarette smoke also leads to (2–3-fold) higher Cd concentrations in the blood and urine compared to individuals who are neither active nor passive smokers [71,72].

The exposure of the general population to Cd may be monitored by evaluating the concentration of this xenobiotic in food and its total daily dietary intake [18,47]. However, due to the difficulty of precisely evaluating the daily intake of Cd, the influence of various factors on its absorption, the uncertainty as to whether a person is an active and/or passive tobacco smoker, and the possibility of additional exposure from sources other than the diet (i.e., the workplace or passive tobacco smoking), calculating the daily intake of this element is not considered a credible method for estimating Cd exposure. Measuring the Cd concentration in the blood and urine is the most reliable method for quantifying the exposure to this xenobiotic because its levels in these biological fluids reflect the exposure from all sources. The blood concentration of this element reflects the current exposure (within the last month), while the concentration in the urine is a more effective biomarker to monitor chronic intoxication [9,27,29,81]. Since Cd is a common contaminant of the environment and food, it is always present in the blood and urine of humans (Table 2 and Table 3). Concentrations of this element below 1 μg/g creatinine in the urine and 0.5 μg/L in the blood are recognized as “normal Cd concentrations” for the general population, defined as very low and safe concentrations resulting from inevitable exposure to low levels in the natural environment and in food [2]. The most recent comprehensive report on worldwide exposure to Cd was published in 2012 [82]. Moreover, there is no global system for monitoring environmental exposure to Cd in areas recognized as unpolluted by this heavy metal. Furthermore, the available data on the current concentrations of Cd in the blood and urine of inhabitants of unpolluted areas are incomplete, and originate from studies conducted in a limited number of countries (Table 3). In addition, the concentration of this element is expressed in various forms (AM, geometric mean (GM), or median), and its values in the urine are not always adjusted for the creatinine concentration (μg/g creatinine), sometimes being expressed as μg/L. Therefore, comparing data between studies is sometimes very difficult.

According to our review of the available data, the Cd concentration in the blood of the general population in industrialized countries worldwide ranges from 0.02 to 4.40 μg/L (0.02–2.88 μg/L in males and 0.02–4.40 μg/L in females), whereas its urinary concentration reaches 0.04–3.39 μg/g creatinine (0.04–2.34 μg/g creatinine in males and 0.09–3.39 μg/g creatinine in females) and 0.01–3.00 μg/L, and is generally higher in females than in males (Table 3). The higher concentration of Cd in the biological fluids of women compared to men may be explained by its higher rate of gastrointestinal absorption in women due to the smaller iron stores in the body and frequent deficiency of this bioelement. The blood and urinary concentrations of Cd in inhabitants of industrialized countries depend on several factors, mainly including smoking habits, age, and the pollution levels in the place of residence (Table 2 and Table 3). Due to the cumulative properties of this xenobiotic, its content in the body increases with age [47,57,83]. Available data in the literature show that the worldwide Cd concentration in the blood of non-smoking individuals reaches 0.09–1.88 μg/L, while in smokers it is higher, ranging from 0.22 to 3.75 μg/L (Table 2) and reaching 7 μg/L in heavy smokers (more than 20 cigarettes/day) [2]. The Cd concentration in the blood and urine increases with the extent of industrialization in the place of residence, as well as the degree of contamination with this xenobiotic [2,21,30,83,84,85,86]. According to the available data, the concentration of Cd in the blood and urine in the general population is lowest in countries such as Sweden and Canada, while the highest levels are found in South Korea and China (Table 3). According to this overview of recently published data, the current Cd exposure levels in industrialized countries worldwide, except for areas recognized as excessively polluted, are low to moderate.

Although the present article is focused on environmental exposure to Cd, one should not ignore that another source of intoxication with this element is the inhalation of airborne Cd particles in the workplace (e.g., in the production of alloys and batteries; the coating, enameling, and smelting of metals; and the printing of textiles) [87,88,89,90,91,92,93,94]. The concentration of Cd in the blood and urine of individuals occupationally exposed to this element exceeds the “normal concentration” of this heavy metal by many times, and is higher than that noted in persons who are not occupationally exposed, reaching 34 μg/L in the blood and 62 μg/g creatinine in the urine [90].

**Table 3:** The current concentration of cadmium (Cd) in the blood and urine of the general population ^a^.

Country	*n*	Expression of Cd Concentration	Cd in the Blood (μg/L)		Cd in the Urine (μg/L) (μg/g Creatinine) ^b^		Reference
			Male	Female	Male	Female	
Argentina	172	Median (range)		0.36 (0.17–1.00)		0.24 (0.01–1.5)	[95]
Bangladesh	72	Median (range)				0.22 (0.01–1.5)	
Canada	10,099	GM (SE)	0.35 (0.01)	0.45 (0.01) ^†^			[73]
	7082	GM (SE)	0.34 (0.01)	0.43 (0.02) *	*0.35 (0.01)*	0.53 (0.01) *	[71]
China	896	Median (P25–P75)	1.34 (0.38–2.88)	0.49 (0.31–0.65) ^NP^	*0.38* *(0.21–0.65)*	*0.42* *(0.23–0.70)* ^NP^	[84]
	78	Median (P25–P75)		1.44 (0.87–2.33)		2.20 (1.42–3.00)	[96]
Ireland	100	Median (P25–P75)			0.3 (0.2–0.6)	0.4 (0.2–0.9)	[97]
South Korea	12,099	GM (95% Cl)	0.76 (0.74–0.77)	1.01 (0.99–1.03) ^NP^			[58]
	643	GM (GSD)	1.10 (1.77)	1.29 (1.78) *	*0.82 (2.04)*	1.04 (2.29) *	[29]
	1907	GM (P95)			*0.82 (2.34)*	*1.36 (3.39)* ^†^	[37]
	3781	Median (P25–P75)			0.42 (0.19–0.77)	0.43 (0.18–0.87)	[98]
Sweden	109	Mean (range)	0.46 (0.02–2.3)	0.54 (0.02–2.9) ^NP^	*0.23* *(0.04–0.80)*	*0.34* *(0.09–1.12)* ^NP^	[30]
Thailand	392	GM (SD)			*0.28 (0.84)*	*0.23 (0.49)* ^NS^	[99]
	81	GM (GSD)	0.9 (2.2)		0.5 (1.9)	1.1 (2.3) ^NP^	[100]
Turkey	30	Median (min–max)		0.34 (0.11–0.84)		0.42 (0.08–0.98)	[101]
USA	3226	GM (SE)	0.49 (0.02)				[102]
	9662	Mean ± SD	0.52 ± 0.58	0.40 ± 0.47			[103]
Denmark	282	GM (95% Cl)			0.123 (0.112–0.350)		[104]
Iceland	203	GM (95% Cl)			0.135 (0.119–0.153)		
Czech Republic	300	GM (95% Cl)			0.132 (0.122–0.142)		
Poland	228	GM (95% Cl)			0.408 (0.369–0.450)		
Croatia	300	GM (95% Cl)			0.175 (0.160–0.192)		
Portugal	295	GM (95% Cl)			0.109 (0.098–0.120)		
France	393	GM (95% Cl)			0.365 (0.340–0.391)		
Luxembourg	210	GM (95% Cl)			0.316 (0.288–0.347)		
Germany	289	GM (95% Cl)			0.199 (0.186–0.213)		
China	50	GM (range)		0.99 (0.23–2.6)			[105]
Croatia	59	GM (range)		0.56 (0.15–4.4)			
Czech Republic	50	GM (range)		0.41 (0.11–2.1)			
Ecuador	25	GM (range)		0.61 (0.25–2.1)			
Morocco	49	GM (range)		0.39 (0.15–1.8)			
Slovakia	52	GM (range)		0.40 (0.17–2.1)			
Slovenia	50	GM (range)		0.49 (0.21–2.2)			
Sweden (north)	35	GM (range)		0.25 (0.08–1.8)			
Sweden (south)	55	GM (range)		0.35 (0.11–2.6)			

GM, geometric mean; GSD, geometric standard deviation; *n*, number of individuals; P25, 25th percentile; P75, 75th percentile; SD, standard deviation; SE, standard error; 95% CI, 95% confidence interval; ^NP^, data regarding the statistical significance of differences were not provided; ^NS^, no statistically significant difference compared to non-smokers; * *p* < 0.05 and ^†^ *p* < 0.01 compared to males; ^a^ based on studies published in the last 10 years; ^b^ values in italics represent Cd concentration in the urine expressed as μg/g creatinine.

## 5. Kidneys as the Main Organ of Cd Accumulation in the Body

After entering the bloodstream, the absorbed Cd binds with thiol groups (sulfhydryl groups, -SH groups) of proteins in the erythrocyte membranes and plasma (mainly with albumins), and most of it is transported with the blood into the liver. In this organ, ions of Cd (Cd^2+^) induce the synthesis of MT and form complexes with this protein (Cd-MT complexes). Some of these complexes are released from the liver into the bloodstream and pass into the tubular fluid [43,52,106]. Moreover, small amounts of this element bound to thiol-containing compounds (e.g., GSH, L-cysteine, L-homocysteine, and N-acetyl-L-cysteine) in the plasma are carried to the kidneys and can be absorbed via the cells of the renal proximal tubules [107].

The main locations of Cd accumulation in both human and animal bodies are the liver and kidneys. During short-term intoxication, Cd is retained mainly in the liver, while long-term exposure results in the accumulation of this xenobiotic mainly in the kidneys, due to their inability to eliminate it from the renal tissues [32,42,108]. The average half-life of Cd in the kidney is 14 years (9–28 years), but some data suggest that it may reach 45 years [18,109]. Thus, the kidney Cd content increases with age, peaking at around 60 years [71,110].

The concentration of this element in the kidneys of the general population (Table 4) has not yet been precisely estimated because of the substantial difficulty of obtaining such data. The only method that allows for the determination of the Cd content in the kidney in vivo, i.e., neutron activation analysis [111], has not been used in epidemiological studies. To our knowledge, the burden of Cd on the kidneys has not been evaluated using this method in humans. Data on the Cd concentration in the kidney usually originate from studies carried out post mortem or in living donors. According to the available data, the Cd concentrations detected in the kidneys of the general population represent a wide range of values, from 1.45 to 93 μg/g wet weight (w.w.) (Table 4). The very limited data from the last 10 years show that the mean concentration of this heavy metal in this organ is 16.0 ± 13.2 μg/g w.w. in subjects aged 37.1 ± 18.7 [112]. The concentration of Cd in the kidneys of individuals occupationally exposed to this xenobiotic [2,88,89,90,94] may be many times higher (150–395 μg/g w.w.) than in the general population (Table 4).

Cd accumulates in the body mainly in the form of complexes with MT. The MT family is a group of cysteine-rich proteins that have a high affinity to various elements, including both necessary and toxic elements, due to the abundance of -SH groups in their cysteine residues. The physiological role of MT is to regulate the metabolism of bioelements such as copper, zinc, and selenium. Furthermore, this protein protects against the toxicity of heavy metals, including Cd, mercury, and lead [56,107,121]. MT binds Cd^2+^ ions in the kidney cells, forming Cd-MT complexes, which are non-toxic; however, their presence in the extracellular space is dangerous [122].

Since data concerning Cd accumulation in the human kidney are highly limited, the retention of this element in the kidney was investigated almost exclusively based on experimental studies conducted using laboratory animals. The available literature contains significant amounts of data on the Cd concentration in the kidneys of animals intoxicated with this element; however, most of these studies considered high exposure levels [106,123,124]. To our knowledge, the only published data on Cd accumulation in the kidney in an experimental model that accurately reflected the current environmental exposure of the general population to this heavy metal originated from our study, which was conducted on rats fed for up to 24 months with a diet containing 1 or 5 mg Cd/kg, corresponding to low or moderate lifetime human exposure, respectively (Table S2) [17]. The study showed that both the low (0.103–0.306 μg Cd/L in the blood and 0.085–0.276 μg Cd/g creatinine in the urine) and moderate (0.584–1.332 μg Cd/L in the blood and 0.284–0.820 μg/g creatinine in the urine) levels of lifelong exposure to Cd led to an increase (up to 100-fold) in the content and concentration of this heavy metal in the renal tissue of rats, and that the accumulation of this xenobiotic was dose- and time-dependent (Table S2). The finding was that under very low and low exposure to Cd (the control group, fed with a diet containing 0.098 mg Cd/kg, and the group administered with 1 mg Cd/kg of feed, respectively), the accumulation of this element in the kidneys of rats increased throughout the experiment and reached a peak after 24 months (0.084 ± 0.036 and 1.98 ± 0.509 μg/g w.w., respectively) (Table S2), when the age of the animals corresponded to the human age of 60 years [125]. This agrees with the observation that in humans, the accumulation of Cd in this organ reaches a peak at around 60 years of age [71,110]. However, the accumulation of this toxic element in the kidneys during moderate exposure reached its peak after 17 months (10.77 ± 1.936 μg/g w.w.), representing approximately 45 human years, before a plateau was reached (Table S2). The extrapolation of our findings regarding Cd accumulation in the kidneys of rats to humans could be inaccurate and should be approached with caution; yet, this study remains the only attempt to explore this process in vivo considering a lifetime exposure level comparable to that currently noted in the general population in industrialized countries. It is important to emphasize that Cd accumulation in the kidney results from both the intensity of its uptake by this organ and the rate of its elimination.

## 6. Cd as a Nephrotoxic Factor

Both acute and chronic intoxication with Cd may result in kidney dysfunction in humans and experimental animals (for a review, see [15,27,88]). Since acute poisoning with this toxic element is very rare nowadays, the risk of acute kidney damage is negligible on a global scale. Chronic occupational [90,93,94] and environmental [7,16,40,126,127] exposure to Cd may cause or contribute to kidney injury; however, the risk of damage to this organ in the general population at the low and moderately low exposure levels that currently occur in many developed and developing countries has not been fully estimated.

The fact that Cd damages the kidneys of humans and experimental animals has been known for a long time. The first cases of this xenobiotic exerting a harmful impact on the kidneys as an outcome of environmental exposure were reported in Japan in the mid-1950s in areas around the Jinzu River, which were polluted by this heavy metal due to the operations of the Kamioka Mine, located upriver [53,128,129,130]. The water of this river was used for both the irrigation of rice fields and fishing. The long-term consumption of food (mainly rice) contaminated with this heavy metal caused chronic Cd poisoning, later called “Itai-Itai” disease. Patients suffering from this disease had a mean Cd concentration of 26.4 μg/g creatinine in the urine and between 10.7 and 46.7 μg/L in the blood [129], while its concentration in the medulla and cortex of the kidney reached 41.6 and 27.8 μg/g w.w., respectively (data presented as GM) [128]. “Itai-Itai” disease first manifested in kidney failure, accompanied by anemia, bone weakening, spinal and leg pains, and deformities, as well as idiopathic bone fractures. Kidney failure, which was a consequence of tubular dysfunction (epithelial cell damage) and glomerular dysfunction, was one of the most dangerous outcomes. This disease resulted in multiple deaths due to kidney failure. The concentration of β2-MG in the urine of “Itai-Itai” disease patients exceeded 1000 μg/g creatinine [129], indicating irreversible kidney damage. The histopathological examination of the renal tissues showed atrophy of the tubular epithelium, accompanied by dilatation of the lumen, the disappearance of renal tubules, and hyalinization and sclerosis in the glomeruli [130].

As in “Itai-Itai” disease patients, analogical changes characterized by damage to the tubules and glomeruli, including irreversible nephropathy, have been found worldwide in the kidneys of individuals chronically exposed to Cd in the workplace [84,121]. Workers employed in a nickel–cadmium battery factory presented Cd concentrations in the blood and urine reaching 10.21 ± 2.671 µg/L (mean ± standard deviation (SD)) and 5.16 µg/g creatinine (median; range: 1.93–8.76 µg/g creatinine), respectively, resulting in damage to the tubules and glomeruli [121]. More recent data show that the present occupational exposure to Cd poses a risk of developing pathological changes in the structure and function of the kidney, such as tubulointerstitial injury, the degeneration of the tubular epithelial cells in the cortex, and microproteinuria [23,89,90,94]. The NOAEL and LOAEL of the Cd concentration in the blood for kidney damage in people occupationally exposed to this heavy metal for 30 years were estimated (based on the concentration of β2-MG in the urine) to be 2.2 and 2.7 µg/L, respectively, while in the case of 40-year exposure, these values reached 1.7 and 2.0 µg/L, respectively [23].

High environmental exposure to Cd (10 µg Cd/L in the blood and higher) resulting in the development of serious kidney damage, as in “Itai-Itai” disease patients, is not found nowadays. However, as mentioned above, epidemiological studies over the years have indicated a risk of kidney injury as an outcome of even low-level exposure [2,54,99,102,131]. The influence of low to moderate exposure to this xenobiotic on renal tissue is described and discussed in detail later in this review.

The revelation of Cd’s damaging impact on the kidneys of “Itai-Itai” disease patients and workers occupationally exposed to this xenobiotic prompted experimental studies using animal models or kidney cell cultures to unveil the mechanisms behind this effect and establish the threshold concentration of Cd in the kidneys, blood, and urine for nephrotoxicity. The toxic effect of this heavy metal in rodent renal tissue (Tables S3 and S4) manifests in numerous defects analogical to those observed in the human kidney, with an identical destruction process, advancing from tubular damage to glomerular disruption. The main histopathological changes in the kidneys of rodents due to Cd exposure are the hypertrophy of epithelial cells, the desquamation of tubular epithelial cells, the dilatation of tubules, and the enlargement of renal glomeruli (Tables S3 and S4) [132,133,134]. Although multiple studies have been conducted regarding the toxic effect of this xenobiotic on the renal tissue of laboratory animals, experiments using models that closely reflect the current lifetime human environmental exposure levels are lacking. Studies on the nephrotoxicity of Cd have mainly been conducted in animal models exposed to moderate, high, and even very high doses of this xenobiotic; furthermore, the exposure routes often do not correspond to those affecting humans (Tables S3 and S4). These studies provided important data on the impact of Cd on the kidney and the possible mechanisms underlying its nephrotoxicity; however, they did not explain the effects of Cd under low-level long-term exposure. Hence, we conducted a study to investigate the damaging impact of Cd on the kidneys and the possible risk of its occurrence in an experimental rat model that accurately reflected lifetime low and moderate human exposure levels (1 and 5 mg Cd/kg of diet for 3–24 months). To our knowledge, the impact of such low chronic exposure to this toxic heavy metal on the kidneys has not yet been investigated in an experimental model, and the findings will be published soon.

The critical concentration of Cd in the renal cortex, endangering 10% of the population, is currently considered to be 50 µg/g w.w. and above [9,24,135]. The results of epidemiological studies suggest that damage to the kidney may occur at lower concentrations of this metal; however, evaluating the threshold level is difficult because epidemiological data on the concentration of this element in renal tissues are lacking. It is important to underline that the risk of damage to the kidney depends on not only the level of exposure to Cd, but also factors such as the exposure duration and chemical form of the xenobiotic, as well as the characteristics of the exposed person (mainly age, sex, diet, and health status), which are recognized as important determinants [2,14,47,63,67,68,126,136].

## 7. Mechanism of Cd Nephrotoxicity

**Figure 2:**
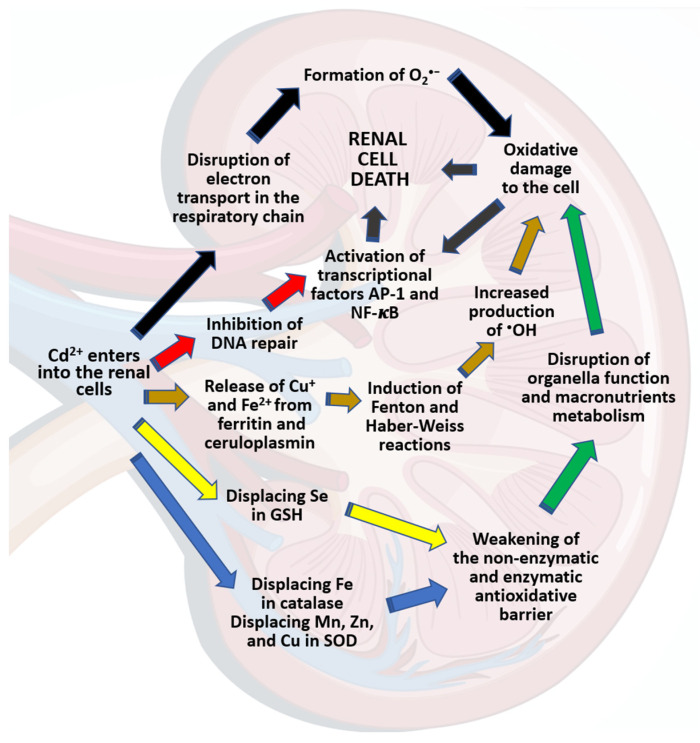
The mechanisms behind the nephrotoxicity of cadmium (Cd). AP-1, activator protein 1; Cd^2+^, cadmium ions; Cu^+^, copper (I) ions; DNA, deoxyribonucleic acid; Fe, iron; Fe^2+^, iron (II) ions; GSH, reduced glutathione; Mn, manganese; NF-κB, nuclear factor kappa-B; O_2_^•−^, superoxide radical; ^•^OH, hydroxyl radical; Se, selenium; SOD, superoxide dismutase; Zn, zinc. This figure was designed using assets from Freepik.com.

The mechanism behind the damaging impact of Cd on the kidney has been the subject of numerous studies conducted using in vivo and in vitro experimental models (Tables S3 and S4) [39,44,107,134,137]. According to our current knowledge, the mechanism is multidirectional (Figure 2) and includes the induction of inflammatory processes; the development of oxidative stress; alterations in cell adhesion; the stimulation of cell proliferation; and the induction of epigenetic changes (damage to deoxyribonucleic acid (DNA), the suppression of the DNA repair capacity, the methylation of genes, and the disruption of gene expression) [24,39,123,134,138,139].

The Cd-MT complexes, released from the liver, are easily filtered through the glomeruli and then reabsorbed by the proximal tubules (segment 1 and segment 2) via endocytosis or decomposed in the epithelial cells of the proximal tubules into Cd^2+^ ions and amino acids [43]. Subsequently, these ions induce the synthesis of MT in the kidneys, bind to it, and accumulate in this form. The Cd-MT complexes are characterized by a short lifetime (about 3 days) [140] and decompose to release Cd^2+^ ions, which further induce the synthesis of MT and bind to this protein. However, the ability of the kidney to biosynthesize MT and accumulate Cd in the form of complexes with this protein is limited, and when Cd^2+^ ions can no longer be detoxified by binding with MT, they begin to bind to -SH groups of other proteins, including structural and functional proteins, and the functional groups of other macromolecules, thus exerting toxic effects [43]. When Cd reaches the kidneys at a high enough concentration that the organ cannot prevent its damaging effects, the epithelial cells malfunction, resulting in injury to the proximal tubules. During low or moderate intoxication with this element, apoptotic or autophagic cell death can occur [44]. If the injury to the cells is severe and widespread, the processes of repair are insufficient, and the necrosis of the cells of the proximal tubules occurs [44]. Despite the research presented in [24,107,124,134], the mechanism of Cd-induced glomerular damage is still unknown. Glomerular damage exacerbates kidney dysfunction.

Numerous data show that Cd-induced kidney damage results from this element’s pro-oxidative properties, which weaken the enzymatic and non-enzymatic antioxidative barriers of cells and increase the production of reactive oxygen species (ROS), thus intensifying the oxidative modifications of cellular macromolecules and damaging cellular organelles (Figure 2) [12,24]. Although this xenobiotic cannot generate free radicals and ROS by itself, it can indirectly generate nitryl, hydroxyl (^•^OH), and superoxide (O_2_^•−^) radicals, which increase the concentration of hydrogen peroxide in cells, allowing the Fenton reaction to take place [24,139]. The generation of ROS also occurs due to a decrease in the cellular concentration of GSH in the nephrons after the disruption of the GSH redox cycle. Cd^2+^ ions replace selenium in GSH biosynthesis, causing the formation of an inactive compound. Cd^2+^ ions can also replace ions of other elements, such as iron (II) (Fe^2+^) and copper (I) (Cu^+^), on the membrane proteins. The release of these transition metal ions via the Fenton reaction intensifies the oxidative processes [137]. A high concentration of ROS, as a result of oxidative stress, damages crucial cellular macromolecules (lipids, proteins, and nucleic acids) and cellular structures (including cellular organelles and cellular membranes), and ultimately leads to cell death, including apoptosis, to which renal tubular cells are highly vulnerable [20,138,141]. A consequence of Cd-induced oxidative stress is the dysfunction of the renal mitochondria, which are the target cellular organelles for this xenobiotic [107]. After penetrating the membranes of mitochondria, Cd^2+^ ions interfere with the electron transport chain and lead to electron leakage and the increased production of ROS; furthermore, they disrupt the course of biochemical processes in the mitochondria, such as respiration and the Krebs cycle [142]. As a result, oxidative stress also activates transcriptional factors such as nuclear factor kappa-B (NF-κB) and activator protein 1 (AP-1), as has been shown in animal studies as well as in renal cell cultures [143]. Metabolomic studies, which have become more common in recent years [138,144,145], could provide a more detailed and precise explanation of the mechanisms behind Cd nephrotoxicity in the future.

Although Cd has not yet been shown to lead to human kidney cancer, its damaging impact on genes, as well as alterations of the expression of microribonucleic acid (microRNA) in renal tissues, have been confirmed in various studies conducted on rats [134]. This heavy metal is known to disrupt important stages of the cellular cycle, such as differentiation, proliferation, progression, DNA synthesis, and apoptosis. Cd also disrupts the processes of DNA repair, stimulates the activation of proto-oncogenes, and increases the methylation of DNA [24]. All of these effects can result in carcinogenic changes in the kidney tissue, as well as other pathological changes in the morphological structure of this organ [146,147].

## 8. Biomarkers of Cd-Induced Kidney Damage

**Figure 3:**
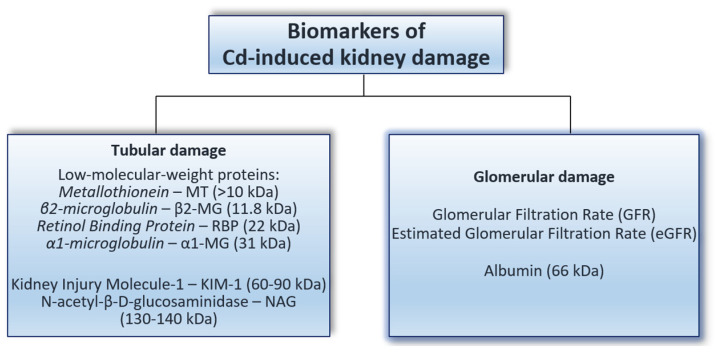
Biomarkers commonly used in the assessment of cadmium (Cd)-induced kidney damage.

In order to monitor exposure to Cd and ensure that it does not exceed the safe threshold for humans, the concentration of this metal in the blood and/or urine is measured [9,80,100]. The blood and urinary concentration of Cd is the most useful biomarker of exposure to this xenobiotic; however, monitoring the exposure level by measuring the Cd concentration in the blood and urine is insufficient due to a lack of feedback on the functional status of the body, including the kidney. The early detection of pathological changes in the kidney as a result of exposure to this toxic element, as well as the proper estimation of the risk of damage to this organ, require sensitive and specific biomarkers of its nephrotoxicity (Figure 3). Appropriate biomarkers enable one to establish the part of the nephron that has been damaged [33,84,91,148]. Due to the inevitability of environmental exposure to Cd in industrialized countries and the available epidemiological data showing that current exposure to this xenobiotic may pose a risk of kidney damage [9,37,47], identifying and applying specific and sensitive biomarkers and properly interpreting changes in their concentrations are necessary for detecting the damaging impact of this xenobiotic on the kidneys at an early stage.

Cd-induced damage to parts of the nephron may be detected using appropriate biomarkers (Figure 3). The determination of β2-MG, α1-MG, RBP, NAG, KIM-1, and albumin in the urine, as well as the assessment of the GFR and eGFR, have been performed in epidemiological studies to evaluate the renal function of people environmentally and occupationally exposed to Cd [4,22,66,91,92,149]. Increased concentrations of β2-MG, α1-MG, RBP, or KIM-1, and the activity of NAG in the urine, indicate tubular damage, whereas a reduced GFR or eGFR and the occurrence of albuminuria are indicators of glomerular damage [33,84,107,148]. It is important to underline that the markers of tubular and glomerular dysfunction used to detect Cd-induced kidney damage are not specific to this xenobiotic. These markers are used for evaluating kidney function in general [34,36,38,46,49,50].

β2-MG and α1-MG are low-molecular-weight proteins that form major histocompatibility complex class I molecules, which are present on the surfaces of almost all nucleated cells and are routinely shed by these cells into the blood. These proteins pass through the glomeruli and, under suitable conditions, are reabsorbed by the proximal tubules; thus, they are present in the urine only in small amounts. However, damage to the renal proximal tubules results in an increase in their concentrations in the urine [44]. A concentration of β2-MG exceeding 300 μg/g creatinine in the urine indicates defective tubular reabsorption [25]. Damage of this degree increases the risk of death due to kidney and urinary tract diseases [150]. Numerous studies have shown that the concentration of β2-MG in the urine rises with increasing exposure to Cd and an increasing concentration of this xenobiotic in the urine [9,37,40,45,145,151,152]. β2-MG is one of the main biomarkers used to evaluate the status of the kidney in “Itai-Itai” disease patients and individuals exposed to Cd occupationally [40,90,91,121,150,153,154]. It is very important to emphasize that the application of β2-MG as a biomarker of nephrotoxicity may be limited due to the degradation of this protein in acidic urine (pH < 5.6), as well as the increase in its concentration in the urine with age and in some diseases [15,154]. The problem of the instability of β2-MG in acidic urine can be avoided by administering bicarbonate to patients before taking urine samples, but this procedure has not yet been carried out in epidemiological studies. The instability of β2-MG in acidic urine can partially explain the discrepancies in the concentration of this protein at similar Cd concentrations in the urine which have been reported by some authors [30,84,155].

Ikeda et al. [156] revealed that an increasing concentration of α1-MG in the urine positively correlated with an increase in the concentration of Cd in both the blood (median = 1.2 μg/L, range = 0.1–6.9 μg/L) and the urine (median = 1.0 µg/g creatinine, range = 0.1–9.6 µg/g creatinine). The cut-off value for this protein is age-dependent: 11.24 μg/g creatinine for ages 18–40 and 19.47 μg/g creatinine for over 40 years of age [157]. Nowadays, α1-MG is rarely used as a marker of Cd nephrotoxicity [96,127].

The use of RBP as an indicator of renal malfunction was proposed in the 1980s, as this protein presents a similar level of sensitivity as a biomarker for this effect to β2-MG [45]. RBP is synthesized mainly in the liver, and its main function is to bind retinol (vitamin A) [158]. Under physiological conditions, this protein is reabsorbed in the renal tubules, but when the reabsorptive function of the tubules is damaged, it appears in the urine. The presence of RBP in the urine is considered to be one of the most sensitive biomarkers for the failure of the proper reabsorptive function of the proximal tubules [91,158]. It is widely used to evaluate the function of the kidney in individuals occupationally exposed to Cd [91,153,154]. However, the usefulness of this marker at Cd concentrations in the urine below 1 μg/g creatinine requires further research [91,151].

The available data from epidemiological studies show that NAG, a cytotoxicity marker enzyme, is a more sensitive biomarker for Cd-induced renal tubular damage than β2-MG and RBP [45]. The activity of NAG in the urine is widely used as a biomarker in studies evaluating the impact of exposure to this heavy metal on the kidneys [81,156,159]. NAG is a lysosomal enzyme abundantly present in the cells of the renal proximal tubules. The activity of this enzyme is low under physiological conditions, and increases during renal tubular cell injury as a result of the growing Cd concentration in the renal tissue. This enzyme occurs in the kidney and urine in two major isoforms, isoenzyme A (NAG-A) and isoenzyme B (NAG-B). NAG-A is released into the urine during the physiological turnover of kidney cells. NAG-B is an intralysosomal membrane-bound enzyme released into the urine upon the disruption of lysosomal membranes. Thus, this isoenzyme is a lesional form of NAG, and is considered to be a highly sensitive indicator of tubular toxicity. However, the activity of total NAG, rather than its isoenzyme B (NAG-B), is commonly assessed in epidemiological studies as a biomarker of Cd nephrotoxicity [45,132]. Although no NAG activity level is currently defined as safe, values over 11 U/g creatinine are considered to indicate renal tubular damage [37].

KIM-1 is one of the most ubiquitous markers of renal failure. It is a transmembrane glycoprotein localized on the epithelial cells of the proximal tubules. Once damage to the epithelial cells of the proximal tubules occurs, KIM-1 is shed into the urine, and, thus, serves as a very sensitive diagnostic indicator of injury to this part of the nephron. The expression of this glycoprotein in a normal kidney is low, but it increases in the injured regions of kidney tubules [160]. Numerous studies, both in humans and experimental animals, have shown an increase in the concentration of KIM-1 due to Cd-induced damage to the proximal tubules [30,44,134,144,161], although some authors have suggested that the usefulness of this marker during low-level exposure to this heavy metal may be limited [28,81]. Recent studies suggest that a KIM-1 concentration of 1.51 μg/L in the urine (median; 0.78–2.55 μg/L) indicates an increased probability of damage to the renal proximal tubules [147,161].

CC16 can also be used to detect renal tubular dysfunction, but the postrenal secretion of this protein from the prostate in men reduces both its specificity and sensitivity. In women, determining the concentration of CC16 in the urine allows for the detection of subtle defects in the proximal tubules that escape notice when other biomarkers are used [162,163]. CC16 is not usually applied in the estimation of kidney function during exposure to Cd, although no evidence is available to prove that CC16 is not useful for this purpose [162].

When assessing the impact of Cd on the function of the renal glomeruli, albuminuria and parameters describing glomerular filtration, such as the GFR and eGFR, are commonly evaluated [30,37,84,164,165]. Albuminuria is the occurrence of an increased amount of albumin in the urine, presenting an albumin concentration (mg/g urine) to creatinine concentration (mg/g urine) ratio over 30. Albumin is the predominant plasma protein normally present in the blood. Under suitable conditions, only trace amounts of this protein occur in the urine; however, under glomerular damage, its concentration in the urine increases [30,149]. The best method for assessing glomerular function is to estimate the rate of glomerular filtration (the amount of fluid filtered from the glomerular capillaries into the Bowman’s capsule per unit of time), expressed as the GFR or eGFR. Studying the GFR involves determining the coefficient of purification of the body using a compound that is filtered in the kidneys, but does not undergo reabsorption in the renal tubules (e.g., creatinine or inulin). The GFR is usually evaluated based on the endogenous creatinine clearance, which represents the amount of creatinine filtered in the glomeruli per unit of time. The eGFR is a mathematically derived entity that is calculated based on an individual’s serum creatinine concentration, age, sex, and race [37]. GFR or eGFR values < 60 mL/min/1.73 m^2^ indicate glomerular damage [37].

The most sensitive biomarkers of nephrotoxicity should be used to estimate the risk of kidney damage due to low or moderate exposure to Cd, allowing for the detection of kidney injury at the earliest stage. Moreover, assessing the impact of low-level Cd exposure on kidney status should be based on determining more than one biomarker of nephrotoxicity, enhancing the possibility of detecting early lesions. Our overview of recent epidemiological studies estimating the impact of current environmental Cd exposure levels on kidney status showed that the most frequently used biomarkers are the β2-MG concentration and NAG activity in the urine, as well as the urinary concentration of albumin and the GFR or eGFR. Due to the interference of Cd in many metabolic pathways in the body, metabolomic studies would likely identify new biomarkers, allowing for a more precise assessment of the risk of kidney damage due to low-level environmental exposure compared to current biomarkers. Potential candidates are compounds such as citrate, creatine, tryptophan, adenine, and uric acid, the values of which in the urine have been found to correlate not only with the concentration of Cd in the urine, but also with commonly used biomarkers of nephrotoxicity, such as the β2-MG concentration and NAG activity [138].

## 9. Risk of Kidney Damage among the General Population at Current Environmental Cd Exposure Levels

**Figure 4:**
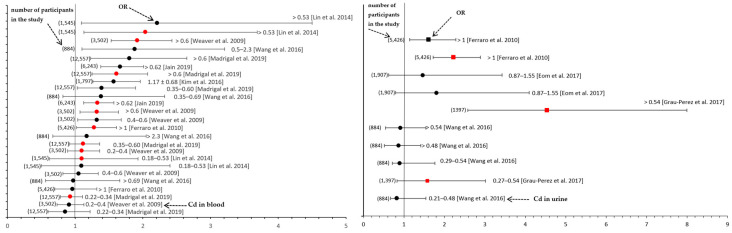
The odds risk (OR) of a reduced estimated glomerular filtration rate (eGFR) (black) and albuminuria (red) dependent on the cadmium (Cd) concentration in the blood (**left**; μg Cd/L) and urine (**right**; square symbols—μg Cd/g creatinine, round symbols—μg Cd/L). Detailed data on the OR values are provided in Table S5 and the following references Eom, S.Y. et al. *Arch. Environ. Contam. Toxicol.* 2017, 73, 401–409 [37]; Ferraro, P.M. et al. *BMC Public Health* 2010, 10, 304 [167]; Grau-Perez, M. et al. *Environ. Int.* 2017, 106, 27–36 [149]; Jain, R.B. *Environ. Sci. Pollut. Res.* 2019, 26, 30112–30118 [5]; Kim, N.H. et al. *J. Korean Med. Sci.* 2015, 30, 272–277 [126]; Lin, Y.S. et al. *Environ. Res.* 2014, 134, 33–38 [66]; Madrigal, J.M. et al. *Environ. Res.* 2019, 169, 180–188 [22]; Wang, D. et al. *Chemosphere* 2016, 147, 3–8 [84]; and Weaver, V. et al. *Am. J. Epidemiol.* 2009, 170, 1156–1164 [168]. The available data show that the threshold Cd concentration for a decreased eGFR and albuminuria is 0.18 μg/L in the blood and 0.27 μg/g creatinine in the urine (Table 5 and Table S5).

Although researchers worldwide have conclusively identified a growing risk of tubular and glomerular kidney impairment due to environmental Cd exposure levels that are considered safe [37,131,155], epidemiological studies exploring the impact of the current environmental Cd exposure levels on kidney status in developed countries are scarce, and the risk of kidney damage has not been well-assessed. Reduced tubular reabsorption and tubular injury have been reported as a result of low-level and moderate environmental exposure to Cd [30,37,45,81,84,156]; however, these changes were not considered clinically relevant. In the present article, the evaluation of whether the current levels of environmental exposure to Cd in industrialized countries pose a substantial risk of clinically relevant kidney damage was based on reliable clinical biomarkers for kidney dysfunction and CKD diagnosis, such as albuminuria, the GFR, and the eGFR, as well as the odds risk (OR) of changes in these parameters. Moreover, available data on biomarkers of tubular damage and the OR of their changes upon low-level and moderate chronic exposure were considered in order to compare the risk of damage to the tubules and glomeruli. To estimate the risk of kidney damage, we considered all available literature data from the last 10 years pertaining to the values of Cd nephrotoxicity biomarkers and the OR of changes in these parameters at Cd concentrations within the range currently found in the general population worldwide (0.02–4.40 μg Cd/L in the blood and 0.04–3.39 μg Cd/g creatinine in the urine; Table 3) (Figure 4, Table 5 and Table S5).

**Table 5:** The link between the concentration of cadmium (Cd) in the urine and biomarkers of tubular and glomerular damage ^a^.

Expression of Data	Cd Concentration in the Urine (μg/g Creatinine)	Biomarkers of Tubular Damage			Biomarkers of Glomerular Damage		Reference
		α1-MG (mg/g Creatinine)	β2-MG (μg/g Creatinine)	NAG (U/g Creatinine)	Albumin (mg/L) (mg/g Creatinine) ^b^	eGFR (mL/min/1.73 m^2^)	
Median (P25, P75)	M: 0.38 (0.21–0.65) F: 0.42 (0.23–0.70)		M: 370 (0.00, 3135) F: 280 (0.00, 2090)	M:10.31 (1.46, 199.09) F: 10.09 (2.16, 48.63)		M: 84.89 (19.30, 204.34) F: 78.56 (19.32, 239.60)	[84]
Median (P5–P95)	2.1 (0.3–5.2)		140 (40–1500)	2.50 (0.09–15.0)	*2.6 (0.5–26.3)*		[45]
GM (95% CI)	M: 0.82 (0.79, 0.86) F: 1.36 (1.31, 1.41)		M: 80.47 (72.22, 88.72) F: 79.86 (74.29, 85.43)	M: 4.17 (3.74, 4.59) F: 4.14 (3.74, 4.54)		M: 91.88 (90.63, 93.13) F: 97.89 (96.79, 98.99)	[37]
Median (IQR)	0.41 (0.195–1.26)		99.8 (71.0–186.80)			109.52 ± 17.43 ^c^	[155]
Mean (range)	0.29 (0.04–1.12)	5.1 (2.0–15.2)	150 (10–1300)	1.95 (0.18–4.88)	*4.8* *(0.69–23.6)*	101 (77–140) 91 (43–178)	[30]
Median (GM)	2.20 (2.10)	1.30 (1.13)		6.95 (6.13)	1.40 (1.42)		[96]
Median (P5–P95)	<2.05 ≥2.05–<3.97	3.82 (0.00–17.27) 4.89 (0.00–19.42)	60 (0.00–5000) 90 (0.00–1760)	6.51 (2.93–62.83) 10.28 (3.69–82.69)	2.84 (0.00–32.37)	66.0 ± 11.3	[164]
Median (min–max)	0.19 (0.01–2.79)	4.13 (0.47–60.1)			6.87 (3.50–11.9)		[127]
Mean (range)	0.11 (0.01–0.52)	2.0 (0.11–31)			*6.3 (1.1–78)*		[166]
Mean	<1.0 1.0–1.9 2.0–4.9		84.65 140.89 115.74	2.12 2.82 2.90			[29]
GM (GSD)	M: 0.5 (1.9) F: 1.1 (2.3)		M: 249.6 (4.0) F: 187.2 (6.6)	M: 5.2 (2.1) F: 4.8 (2.3)			[100]
Mean (SD)	1.08 (1.98)		51.4 (2.64)	4.01 (2.78)			[131]
Median (P25–P75)	2.25 (1.20–5.10) ^d^	0.31 (0.07–6.49)	110 (70–2800)	5.10 (3.30–7.25)	2.86 (0.46–7.03)		[81]

The values of the urine parameters that indicate renal damage are: α1-MG—11.24 μg/g creatinine, β2-MG—300 μg/g creatinine, and NAG—11 U/g creatinine for tubular damage; and albumin—30 mg/g creatinine (or 20 mg albumin/L) and eGFR ≤ 60 mL/min/1.73 m^2^ for glomerular damage. F, female; IQR, interquartile range; GM, geometric mean; GSD, geometric standard deviation; M, male; P5, 5th percentile; P75, 75th percentile; P95, 95th percentile; SD, standard deviation; 95% CI, 95% confidence interval; ^a^ based on studies conducted in the last 10 years; ^b^ values in italics represent albumin concentration in the urine expressed as mg/g creatinine, ^c^ mean ± SD; ^d^ μg/L.

Evaluating the risk of kidney damage at the current environmental Cd exposure levels based on the available data is difficult, because the data are limited; the impact of Cd is often assessed using different biomarkers of nephrotoxicity; the OR of changes in certain parameters is not always provided; the urinary concentration of Cd is not adjusted for the creatinine concentration (sometimes expressed as μg/L) in every study; and some studies only determine the Cd concentration in the blood. Various authors have evaluated the impact of the current Cd exposure levels on kidney status based on biomarkers such as β2-MG, α1-MG, NAG, RBP, albuminuria, and the GFR or eGFR, as well as estimating the OR of changes in these parameters (Figure 4, Table 5 and Table S5) [4,45]. However, studies determining whether the current levels of environmental exposure to this xenobiotic pose a risk of kidney damage most often considered β2-MG, NAG, albuminuria, and the GFR or eGFR (Table 5 and Table S5, Figure 4).

Regardless of the biomarker of tubular or glomerular damage which is used, an OR > 1 indicates an increased risk of damage to the respective part of the nephron. Thus, the OR is an effective parameter to estimate the risk of Cd nephrotoxicity. Although the proximal tubule is the critical part of the nephron under exposure to Cd, researchers have focused on evaluating the OR of glomerular damage due to current levels of environmental exposure to Cd rather than the OR of tubular damage, because damage to the glomeruli is clinically relevant and may result in the development of CKD. In most studies, even if biomarkers of tubular damage were determined, the ORs of changes in these parameters were not calculated [45,81,84,96,127,131,155,164]. Thus, the risk of tubular injury at low to moderate Cd exposure in the general population has not been thoroughly assessed; however, the available data [37] allow us to conclude that this risk is higher than that of glomerular damage and occurs at a lower concentration of Cd in the blood and urine than that at which glomerular damage occurs.

The detailed overview of the available data suggested that the current levels of environmental exposure to Cd in developed countries have increased the risk of damage to both the tubules and glomeruli, as OR values for changes in the biomarkers of Cd nephrotoxicity exceeding 1 and reaching 13.29 have been noted in this Cd concentration range in the blood and urine of the general population (Figure 4, Table S5) [4,45]. It is important to emphasize that in some studies [45], an OR exceeding 1 was found for more than four biomarkers of kidney status (β2-MG, α1-MG, NAG, albuminuria, and the eGFR), providing clear evidence of an increased risk of damage to both tubules and glomeruli. The only available data pertaining to the OR of changes in the biomarkers of tubular damage under low-level exposure to Cd referred to β2-MG and NAG [37]. Eom et al. [37] revealed that at Cd concentrations in the urine ranging from 0.87 μg/L to 1.55 μg/L, the ORs of increases in the β2-MG concentration and NAG activity reached 4.07 (1.35–12.24) and 1.47 (0.80–2.70), respectively, while the OR of decreased eGFR was 1.46 (0.62–3.43). The finding that the ORs of changes in the β2-MG concentration and NAG activity exceeded 1 at urine Cd concentrations between 0.87 and 1.55 μg/L showed that people presenting such concentrations of this toxic element in their urine are at a higher risk of tubular damage.

Grau-Perez et al. [149] observed a reduced risk of developing albuminuria when the Cd concentration in the urine did not exceed 0.27 µg/g creatinine, whereas twice this concentration (0.54 µg/g creatinine) corresponded to a threefold higher OR for albuminuria, with the risk being positively correlated with the Cd concentration in the urine. Other studies also showed that the risk of albuminuria rose significantly for a urine Cd concentration exceeding 1 µg/g creatinine [167]. According to studies carried out on individuals environmentally exposed to Cd, the risk of albuminuria increased when the Cd concentration was higher than 0.18 μg/L in the blood and 0.27 μg/L in the urine [22,66,167,168]. The Cd concentration in both the blood and urine seems to be a very useful parameter for evaluating the risk of developing albuminuria. An increased OR of albuminuria has been noted at blood Cd concentrations ranging from 0.18 μg/L to 1 μg/L [66,84] (Figure 4, Table S5).

Cd has been shown to impair the ability of the kidney to conduct proper glomerular filtration (evaluated according to the eGFR) at concentrations in the blood of 0.18 μg/L or higher, and the risk of a reduced eGFR correlates positively with the blood Cd concentration [66,84]. A similar relationship has been shown in other studies [124,164,165,167]. Ferraro et al. [167] reported that the OR of a reduced eGFR reached 1.48 at a Cd concentration > 1 μg/L in the blood and urine. A Korean study [126] found that the OR of a reduced eGFR was 1.57 at a blood Cd concentration of 1.17 ± 0.68 μg/L. These changes in the biomarkers of Cd nephrotoxicity and the ORs of these changes at urine Cd concentrations currently found in the general population (Figure 4, Table 3 and Table 5) imply that environmental exposure to this heavy metal is a threat to kidney health. Importantly, the ORs of albuminuria and a decreased eGFR were reported to increase at Cd concentrations in the blood and urine starting from 0.18 μg/L and 0.27 μg/g creatinine, respectively, showing that the risk of glomerular damage also occurs at relatively low Cd concentrations (within the range measured in the general population; Figure 4, Table S5). Unfortunately, biomarkers of tubular damage were not determined in these studies [5,125,149,169]. However, Eom et al. [37] revealed that the OR of changes in β2-MG was higher that of glomerular damage (eGFR) at the same urine Cd concentration, confirming the higher risk of tubular injury than glomeruli injury.

The available data show that the NOAELs of the Cd concentration in the blood and urine for clinically relevant kidney damage (glomerular dysfunction expressed as albuminuria and decreased eGFR) due to environmental exposure are 0.18 μg/L and 0.27 μg/g creatinine, respectively, whereas the LOAELs are >0.18 μg/L and >0.27 μg/g creatinine, respectively (Figure 4, Table 5 and Table S5). Since the blood and urinary concentrations of Cd found in the general population worldwide range from 0.02 to 4.40 μg/L and from 0.04 to 3.39 μg/g creatinine (Table 3), respectively, the current levels of environmental exposure to this heavy metal in industrialized countries may pose a substantial risk of CKD or at least contribute to its development. This conclusion was based on relatively limited epidemiological data; however, in the available literature, only these data were relevant for the current low and moderate levels of environmental exposure to Cd. To strengthen the above conclusion, it is important to underline that each study exploring the impact of low to moderate environmental Cd exposure on kidney status revealed an increased risk of kidney damage. Therefore, the claim that Cd should be considered an environmental risk factor for CKD is reasonable.

## 10. Exposure to Cd as a Factor Increasing the Risk of Kidney Damage Due to Other Causes

As our review of the available data revealed that the current levels of environmental exposure to Cd pose a risk of kidney damage, it is very important to underline that such exposure could exacerbate pathological changes in the kidney resulting from the action of other nephrotoxic factors; worsen the course of certain disease states; and potentiate the health effects of other xenobiotics [4,92,170,171,172]. Co-exposure to Cd and other compounds with and without nephrotoxic properties, such as microplastics [173] and heavy metals including chromium [174], lead [175,176], mercury [87], uranium [177], and thallium [7], was found to pose a more substantial threat to the human kidney than Cd alone. Jain [5] reported that co-exposure to Cd, mercury, and lead increased the risk of kidney malfunction by up to twofold compared to exposure to Cd alone. Occupational exposure to mixtures of Cd and lead or Cd, lead, and chromium resulted in more serious damage to the kidneys than exposure to each metal individually [4,6,52,153], resulting in a higher risk of a decrease in the eGFR (OR: 7.9, 95% CI: 0.9–67.2) [4] and an increase in early renal biomarkers such as urinary NAG activity and albuminuria [153]. The results of an experimental study by Riaz et al. [124] suggested that kidney injury due to co-exposure to Cd and other toxic elements (lead, manganese, and arsenic) could be amplified in a chronic disease such as diabetes. Co-exposure disrupted the kidneys more seriously than Cd alone, resulting in more substantial damage to the renal cells (tubular degeneration, fibrosis, and the vacuolation of cells) and the weakening of the antioxidative barrier.

## 11. Conclusions and Outlook

This overview allowed us to estimate the NOAELs of the Cd concentration in the blood and urine for clinically relevant kidney damage (glomerular dysfunction) in the general population to be 0.18 μg/L and 0.27 μg/g creatinine, respectively, whereas the LOAELs were estimated to be >0.18 μg/L and >0.27 μg/g creatinine, respectively. The current levels of environmental exposure to Cd in industrialized countries have resulted in Cd concentrations in the blood and urine of 0.02–4.40 μg/L and 0.04–3.39 μg/g creatinine, respectively, within the range at which an increased risk of damage to the kidney tubules and glomeruli has been observed (above 0.18 μg/L and 0.27 μg/g creatinine, respectively). Thus, this level of exposure may pose a substantial risk of damage to the kidneys, potentially causing, or at least contributing to, the development of CKD. Moreover, β2-MG and NAG in the urine appear to be sensitive biomarkers, allowing for the detection of disturbances in the proximal tubules, whereas albuminuria and the glomerular filtration rate (evaluated as either the GFR or eGFR) are early markers of glomerular damage during low-level exposure to this heavy metal.

This paper shed new light on the possible causative factors of CKD, one of the main worldwide health problems. Even low levels of environmental exposure to Cd may increase the risk of not only tubular damage, but also clinically relevant disorders such as glomerular dysfunction in the general population. Thus, this xenobiotic should be considered as a possible causative factor of kidney diseases with unknown etiologies. Environmental exposure to Cd may be responsible for some etiologically unspecified cases of CKD among the inhabitants of industrialized countries worldwide. Hence, the clinical histories of patients presenting with CKD of an unknown etiology should include their histories of exposure to Cd; furthermore, in such cases, the Cd concentration in the blood and/or urine should be determined, as this may be helpful. Considering the credibility of the evidence that the current levels of environmental exposure to Cd may pose a risk of kidney damage in the general population, re-establishing lower “normal Cd concentrations” in the blood and urine should be considered. The overview presented herein showed that exposure to Cd in industrialized countries should be carefully monitored, and further studies should be conducted to more accurately assess the global risk of kidney damage due to environmental Cd exposure. The evidence that Cd poses a risk of kidney damage at the current levels of exposure in numerous countries suggests that environmental exposure to this toxic metal is an important public health problem. Thus, protective strategies, such as decreasing the Cd pollution in the environment, increasing the efficiency of Cd absorption from the gastrointestinal tract, and enhancing the body’s ability to defend against the toxic effects of this element, should be sought out and implemented.

## Figures and Tables

**Table 1:** The data search strategy regarding the impact of the current environmental exposure to cadmium (Cd) on the kidneys.

Database	Total Number of Articles Found (Published in 2013–2023)	Number of Articles Excluded (Duplicates, Papers Out of Our Scope, or Papers Older than 10 Years)	Number of Articles Included in This Study
Pubmed	3971	3896	75
Scopus	8571	8546	25
Elsevier	3064	3041	23
Taylor & Francis Online	2491	2482	9

**Table 2:** The concentration of cadmium (Cd) in the blood and urine of tobacco smokers compared to people who have never smoked ^a^.

Country	*n*	Expression of Cd Concentration	Cd in the Blood (μg/L) and Urine ^b^ (μg/g Creatinine)		Reference
			Smokers	Non-Smokers	
Canada	10,099	GM (SE)	1.63 (0.06) ^†^ *0.56 (0.02)* ^†^	0.22 (0.01) *0.33 (0.01)*	[73]
	144	GM (P95)	1.62 (3.75) * 0.467 (1.21) *	0.265 (1.88) *0.333 (0.937)*	[74]
Iran	140	Mean (IQR)	0.87 (0.67–1.31) ^NS^	0.81 (0.59–1.30)	[75]
Serbia	81	Mean ± 95% Cl	2.41 ± 0.04 ^‡^	0.67 ± 0.04	[76]
South Korea	200	Mean (SD)	1.67 (0.68) ^NP^	0.83 (4.23)	[77]
	4744	GM (SD)	1.06 (0.02) ^‡^	0.89 (0.01)	[78]
Sweden	4304	Median (P5, P95)	1.00 (0.22–2.46) ^NP^	0.20 (0.09–0.46)	[79]
USA	2325	GM (95% Cl)	1.17 (0.77–1.81) ^NP^	0.86 (0.54–1.36)	[80]
	6761	GM (95% Cl)	1.02 (0.97–1.06) ^‡^ *0.39 (0.36–0.41)* ^‡^	0.24 (0.24–0.25) *0.20 (0.19–0.21)*	[57]

GM, geometric mean; IQR, interquartile range; *n*, number of individuals; P5, 5th percentile; P95, 95th percentile; SD, standard deviation; SE, standard error; 95% CI, 95% confidence interval; ^NS^, no statistically significant difference compared to non-smokers; ^NP^, data regarding the statistical significance of differences were not provided; * *p* < 0.05, ^†^ *p* < 0.01, and ^‡^ *p* < 0.001 compared to non-smokers; ^a^ based on studies published in the last 10 years; ^b^ values in italics represent Cd concentration in the urine.

**Table 4:** The concentration of cadmium (Cd) in the kidneys of different populations non-occupationally exposed to this heavy metal.

Region (*n*)	Expression of Cd Concentration	Cd Concentration in the Kidney (μg/g w.w.)		Reference
Australia (61)	Mean ± SD	15.45 ± 14.04		[108]
Czech Republic (70)	Mean (95% CI)	28.7 (6.61–93.0)		[113]
Greenland (95)	Mean ± SD	15.97 ± 9.26		[114]
Japan (71)	GM ± GSD	Male, cortex: 72.1 ± 1.7	Female, cortex: 83.9 ± 2.2	[115]
		Male, medulla: 18.3 ± 2.2	Female, medulla: 24.5 ± 2.1	
Japan (41)	GM ± SD	Cortex: 82.7 ± 1.99	Medulla: 36.1 ± 1.99	[19]
Norway (28)	Mean (95% CI)	20.5 (3.74–62.16)		[116]
Poland (99)	Mean ± SD	16.0 ± 13.2		[112]
South Korea (150)	Mean ± SD	35 ± 18		[117]
Spain (78)	Mean (95% CI)	10.8 (6.1–20.2)		[118]
Spain (20)	Mean	21.15		[119]
Sweden (10)	Median (95% CI)	5.18 (2.29–29.99)		[41]
Sweden (109)	Median (range)	12.9 (1.50–55.0)		[120]
		Male: 10.9 (1.6–32.0)	Female: 14.7 (1.50–55.0)	

GM, geometric mean; GSD, geometric standard deviation; *n*, number of individuals; SD, standard deviation; w.w., wet weight; 95% CI, 95% confidence interval.

## Data Availability

Not applicable.

## Supplementary Materials

The following supporting information can be downloaded at: https://www.mdpi.com/article/10.3390/ijms24098413/s1. References [5,17,22,37,66,72,84,126,146,149,167,168,169,178,179,180,181,182,183,184,185,186,187,188,189,190,191] are cited in the supplementary materials.

## Abbreviations

α1-MG, α1-microglobulin; AM, arithmetic mean; β2-MG, β2-microglobulin; b.w., body weight; CC16, Clara cell protein 16; Cd, cadmium; Cd^2+^, cadmium ions; Cd-MT, cadmium–metallothionein complex; CKD, chronic kidney disease; Cu^+^, copper (I) ions; DNA, deoxyribonucleic acid; eGFR, estimated glomerular filtration rate; Fe^2+^, iron (II) ions; GFR, glomerular filtration rate; GM, geometric mean; GSH, reduced glutathione; KIM-1, kidney injury molecule-1; LOAEL, lowest observed adverse effect level; MT, metallothionein; NAG, N-acetyl-β-D-glucosaminidase; NAG-A, N-acetyl-β-D-glucosaminidase isoenzyme A; NAG-B, N-acetyl-β-D-glucosaminidase isoenzyme B; NF-κB, nuclear factor kappa-B; NOAEL, no observed adverse effect level; O_2_^•−^, superoxide radical; OR, odds risk; ^•^OH, hydroxyl radical; PTMI, provisional tolerable monthly intake; PTWI, provisional tolerable weekly intake; RBP, retinol binding protein; ROS, reactive oxygen species; SD, standard deviation; -SH group, thiol group (sulfhydryl group); w.w., wet weight.
