# Supplementary material for: Current Levels of Environmental Exposure to Cadmium in Industrialized Countries as a Risk Factor for Kidney Damage in the General Population: A Comprehensive Review of Available Data
Source: Int J Mol Sci. 2023 May 8;24(9):8413. doi: 10.3390/ijms24098413 (PMC10179615; doi:10.3390/ijms24098413)
Supplement: Supplementary file 1 [file ijms-24-08413-s001.zip › ijms-2323298-supplementary.pdf]

International Journal of *Molecular Sciences*

MDPI

Supplementary Materials

Current Levels of Environmental Exposure to Cadmium in Industrialized Countries as a Risk Factor for Kidney Damage in the General Population: A Comprehensive Review of Available Data

Nazar M. Smereczański * and Małgorzata M. Brzóska *

* Department of Toxicology, Medical University of Bialystok, Adama Mickiewicza 2C street, 15-222 Bialystok, Poland
Correspondence: nazar.smereczanski@umb.edu.pl (N.M.S.), malgorzata.brzoska@umb.edu.pl (M.M.B.)
Tel.: +48-85-7485604 (M.M.B. & N.M.S.); Fax: +48-85-7485834 (M.M.B. & N.M.S.)

Table S1. Etiological factors for kidney damage.

**Etiological Factors of Kidney Damage**			
Illnesses	Medicines		Other factors
· hypertension<br>· diabetes<br>· sepsis<br>· liver failure<br>· obesity<br>· glomerular disease<br>· polycystic kidney disease	· polymixin A<br>· bacitracin<br>· phenacetin<br>· acetaminophen (paracetamol)<br>· cisplatin<br>· cyclosporine<br>· ifosfamide<br>· pemetrexed<br>· gentamycin<br>· kanamycin<br>· streptomycin	· tobramycin<br>· colistin<br>· amphotericin B<br>· foscarnet<br>· adefovir<br>· cidofovir<br>· tenofovir<br>· iopromide<br>· tacrolimus<br>· pamidronate<br>· zoledronic acid	· xenobiotics other than medicines: heavy metals (cadmium, lead, mercury, arsenic, chromium, bismuth, nickel), alcohols and glycols (ethyl alcohol, methyl alcohol, ethylene glycol)<br>· toxins (amanitin)<br>· components of tobacco smoke

Table S2. The accumulation of cadmium (Cd) in the kidney in an experimental rat model of human environmental exposure to this heavy metal[a].

Cd Content (μg) or Concentration (μg/g w.w.)	Exposure Duration				Time-related Changes
	3 Months	10 Months	17 Months	24 Months	
	Control				
Content	0.0682 ± 0.0060	0.0912 ± 0.0056	0.1078 ± 0.0059	0.2271 ± 0.0297	3–10*, 3–17‡, 3–24‡ 10–24‡, 17–24*
Concentration	0.0375 ± 0.0102	0.0497 ± 0.0079	0.0467 ± 0.0085	0.0844 ± 0.0357	3–24†, 10–24†, 17–24†
	1 mg Cd/kg of feed (daily Cd intake: 37.50–84.88 μg/kg b.w.)				
Content	0.6340± 0.0369***	2.1794± 0.1202***	2.8677± 0.3347***	5.247± 0.4784***	3–10‡, 3–17‡, 3–24‡ 10–24‡, 17–24†
Concentration	0.3495± 0.0601***	1.103± 0.1968***	1.213± 0.3763***	1.981± 0.5089***	3–17‡, 3–24‡, 10–24†
	5 mg Cd/kg of feed (daily Cd intake: 196.69–404.76 μg/kg b.w.)				
Content	2.562± 0.1454***	10.16 ± 0.427***	25.23 ± 1.678***	22.18 ± 0.971***	3–10‡, 3–17‡, 3–24‡ 10–17‡, 10–24‡
Concentration	1.362± 0.2254***	4.788± 0.5586***	10.77± 1.9360***	8.009± 0.8918***	3–10†, 3–17‡, 3–24† 10–17†, 10–24†

Data is mean ± standard error (SE) for 8 rats, except for 7 animals in the group maintained on the diet containing 1 mg Cd/kg for 24 months. *** $p < 0.001$ compared to the control group at the same time point; time-related changes: * $p < 0.05$, † $p < 0.01$, ‡$p < 0.001$; w.w., wet weight, b.w., body weight. [a] prepared based on Brzóska et al. [1].

Table S3. The effect of oral exposure to cadmium (Cd) on the kidneys of experimental animals[b].

Dosage, Form, and Time of Exposure to Cd	Experimental Model	Changes in the Morphological Structure of the Renal Tissue	Changes in Various Parameters in the Renal Tissue or Serum	Reference
100 mg Cd/L of drinking water, 2 weeks	Male Wistar rats	– intertubular congestion<br>– loss of the brush border<br>– dilatation of convoluted tubules		[2]
200 mg $CdCl_2$/kg b.w. /day in drinking water, 12 weeks	Male rabbits		– ↑ renal expression of the apoptotic (Caspase3), proliferation (MKI67), proto-oncogene (C–fos), and antioxidant (GST) genes<br>–↓ renal expression of anti-apoptotic (Bcl2) genes	[3]
5 mg $CdCl_2$/kg of feed, 30 days	Male Wistar rats	– congestion of the cortical blood vessels<br>–focal replacement of the renal parenchyma with numerous lymphocytes infiltrates<br>–dilation of glomeruli		[4]
15 mg $CdCl_2$/kg b.w./day, 5 weeks	Male Wistar rats	– disruption in the organization of the renal glomeruli and tubules	– ↑ DNA damage in the kidney	[5]
6.3 mg $Cd(NO_3)_2$/kg b.w./day, single dose	Male Wistar rats	– swelling with thickened blood vessel<br>– fatty vacuole<br>– fatty infiltrate<br>– lymphocyte aggregate and infiltration	– ↓ SOD, ↓ CAT, and ↓ GPx activities in the kidney	[6]
5 mg $CdCl_2$/kg b.w./day, 5 weeks	Male Sprague–Dawley rats	– tubular necrosis	– ↓ SOD, ↓ CAT, and ↓ GPx activities in the kidney<br>– ↑ creatinine and ↑ LDH concentrations in the serum<br>– ↓ total thiol concentration in the kidney	[7]
8.8 mg $CdCl_2$/kg b.w./day, 20 days	Pregnant female Sprague–Dawley rats	– hydropic degeneration of the cytoplasm<br>– deterioration of the nuclei of the lining cells of PT and DT in both maternal and fetal kidney		[8]
0.685 mg $CdCl_2$/L of drinking water, 90 days	8 weeks old C57BL mice	– severe vascular degeneration and necrosis of renal tubules with glomerular deterioration		[9]

CAT, catalase; $CdCl_2$, cadmium chloride; $Cd(NO_3)_2$, cadmium nitrate(V); DT, distant tubule; GPx, glutathione peroxidase; GSH, reduced glutathione; GST, glutathione S-transferase; LDH, lactate dehydrogenase; MKI67, a marker of proliferation KI67; PT, proximal tubule; SOD, superoxide dismutase. [b] based on the studies published within the last 10 years.

Table S4. The effect of exposure to cadmium (Cd) via routes other than oral on the kidneys of experimental animals[b].

Dosage, Form, Route, and Time of Exposure to Cd	Experimental Model	Changes in the Morphological Structure of the Renal Tissue	Changes in the Various Parameters in the Renal Tissue, Blood/serum, or Urine	Reference
10 mg $CdCl_2$/kg b.w./day, s.c., 15 and 30 days	Male Wistar rats	mononuclear cell infiltration, interstitial congestion around the glomeruli with wide lumen, pyknotic nuclei, high dilatation in intertubular blood vessels impacted with hemolysed blood and cellular infiltration, congestion and inflammation around glomeruli, degenerated glomeruli with wide space and detached basement membrane, DT with wide lumen, deformed PT with detached brush border, degeneration and hyalinization of glomerular tuft, tubular degeneration, tubular and tubulointerstitial necrosis	– ↑ LOOH, ↑ MDA, and ↑ PC concentrations in the kidney, ↑ TOS, ↑ OSI<br>– ↑ expression of inflammatory markers: Hsp70, COX2, and TNFα in the kidney<br>– ↓ TAS, ↓ activities of SOD and CAT, ↓ GSH concentration in the kidney	[10]
0.6 mg $CdCl_2$/kg b.w., s.c., 5 days/week, 12 weeks	Male Sprague–Dawley rats	fibrosis of the tubules	– ↑ KIM–1 and ↑ β2-MG concentrations and ↑ NAG activity in the urine	[11]
6.5 mg $CdCl_2$/kg b.w./day, i.p., 5 days	Male Wistar rats	moderate to severe inflammation and widespread degeneration of cells, cytoplasmic vacuolization, congested glomeruli, severe apoptosis, karyomegaly, and hyperchromatic nuclei in the tubular epithelial cells	– ↑ MDA concentration in the kidney<br>– ↓ SOD, ↓ CAT, and ↓ GPx, activities in the kidney	[12]
2 mg $CdCl_2$/kg b.w./day, i.p., 4 weeks	Male Wistar rats	severe tubular necrosis and apoptosis, vacuolization, degeneration	– ↑ urea nitrogen concentration in the blood<br>– ↑ creatinine concentration in the serum<br>– ↓ creatinine clearance<br>– ↓ NO, ↓ protein thiols, ↓ free thiols, and ↓ total thiols concentrations and ↓ CAT and ↓ SOD activities in the kidney	[13]
3 mg $CdCl_2$/kg b.w./day, i.p., 7 days	BALB/c mice	cloudy swelling of tubular cells, narrow renal tubules, and fibrosis	– ↓ SOD activity and ↓ GSH concentration in the kidney<br>– ↑ ROS generation and ↑MDA concentration in the kidney	[14]
1 mg $CdCl_2$/kg b.w./day, i.p., 2 weeks	Male Sprague–Dawley rats		– ↑ creatinine concentration in the urine<br>– ↑ ADA, ↑ TNFα, ↑ IL–6, and ↑ IL–10 concentrations in the kidney	[15]
1 mg $CdCl_2$/kg b.w./day, i.p., 5 weeks	Wistar rats	vacuolization of epithelial lining renal tubules, congestion and atrophy of glomerular tufts, and distension of Bowman's space	– ↓ SOD, ↓ CAT, and ↓ GPx, activities in the kidney	[16]
4 mg $CdCl_2$/kg b.w./day, i.v., 2 weeks	Male Wistar rats	tubular degeneration, necrosis and severe renal cortical congestion	– ↑ lipid peroxidation in the kidney<br>– ↓ GSH concentration, ↓ AST, ↓ ALT, ↓ SOD, ↓ CAT and ↓ GPx activities in the kidney	[17]

ADA, renal adenosine deaminase; ALT, alanine aminotransferase; AST, aspartate aminotransferase; β_2-MG, β2-macroglobulin; CAT, catalase; $CdCl_2$, cadmium chloride; COX2, cyclooxygenase 2; DT, distant tubule; GPx, glutathione peroxidase; GSH, reduced glutathione; GST, glutathione S-transferase; Hsp70, heat shock protein 70; IL-6, interleukin 6; IL-10, interleukin 10; i.p., intraperitoneally; i.v., intravenously; KIM-1, kidney

injury molecule-1; LOOH, lipid peroxides; MDA, malondialdehyde; NAG, N-acetyl-β-D-glucosaminidase; NO, nitrogen(II) oxide; OSI, oxidative stress index; PC, protein carbonyls; PT, proximal tubule; ROS, reactive oxygen species; s.c., subcutaneously; SOD, superoxide dismutase; TAS, total antioxidative status; TNFα, tumor necrosis factor α; TOS, total oxidative status. [b] based on the studies published within the last 10 years.

Table S5. The odds risk (OR) of decreased estimated glomerular filtration rate (eGFR) and albuminuria due to low-level environmental exposure to cadmium (Cd).

**Country**	**n**	**Cd in the Blood (μg/L)**	**OR[c] (95% Confidence Interval)**		**Cd in the Urine (μg/L or *μg/g Creatinine*)**	**OR (95% Confidence Interval)**		**Reference**
			**Decrease in eGFR**	**Albuminuria**		**Decrease in eGFR**	**Albuminuria**	
China	884	Men:			Men:			[18]
S and NS		< 0.5	1.00		< 0.29	1.00		
		0.5–2.3	1.88		0.29–0.54	0.89		
			(1.10–3.21)			(0.71–1.77)		
		> 2.3	1.17		> 0.54	0.91		
			(0.67–2.05)			(0.54–1.54)		
		Women:			Women			
		< 0.35	1.00		< 0.21	1.00		
		0.35–0.69	1.38		0.21–0.48	0.82		
			(0.82–2.32)			(0.67–1.54)		
		> 0.69	0.97		> 0.48	0.86		
			(0.56–1.66)			(0.52–1.42)		
USA	3502	< 0.2	1.00	1.00				[19]
S and NS		0.2–0.4	0.91	1.10				
			(0.73–1.13)	(0.89–1.36)				
		0.4–0.6	1.05	1.32				
			(0.82–1.34)	(1.07–1.64)				
		> 0.6	1.32	1.92				
			(1.04–1.68)	(1.53–2.43)				
USA	12577	0.22–0.34	0.85	0.93				[20]
S and NS			(0.60–1.22)	(0.78–1.11)				
		0.35–0.60	1.39	1.12				
			(1.03–1.89)	(0.92–1.36)				
		> 0.60	1.80	1.61				
			(1.23–2.65)	(1.25–2.07)				
USA	5426	< 1	1.00	1.00	*< 1*	1.00	1.00	[21]
S and NS		1 <	0.96	1.28	*> 1*	1.61	2.23	
			(0.70–1.32)	(1.02– 1.61)		(1.14–2.28)	(1.72–2.89)	
USA	1545	< 0.18	1.00	1.00				[22]
S and NS		0.18–0.53	1.09	1.10				
			(0.50–2.40)	(0.62–1.93)				
		> 0.53	2.21	2.04				
			(1.09–4.50)	(1.13–3.69)				
USA	6243	> 0.62	1.663 (1.38–2.01)	1.329				[23]
S and NS				(1.123 – 1.573)				
Spain	1397				*< 0.27*		1.00	[24]
S and NS					*0.27–0.54*		1.58	
					> 0.54		(0.83–3.02)	
							4.54	
							(2.58–8.00)	
South Korea	1797	1.17±0.68	1.57					[25]
S and NS			(1.26–1.96)					
Taiwan	658	< 0.8	1.00					[26]
S and NS		0.8–1.3	2.40					
			(1.14–5.07)					
		> 1.3	6.37					
			(3.05–13.29)					
South Korea	1907				< 0.87	1.00 (Ref.)		[27]
S and NS					0.87–1.55	1.46		
					> 1.55	(0.62–3.43)		
						1.80		
						(0.79–4.10)		

Albuminuria, the albumin/creatinine ratio in the urine > 30; decrease in eGFR, eGFR < 60 mL/min/1.73 m^2. NS, non-smokers; S, smokers; OR, odds risk. [c] The OR represents the odds of an outcome during a particular level of exposure, compared to the odds of the outcome occurring in the absence of this exposure. The OR > 1 indicates an increased risk of the appearance of an effect.

Reference

1. Brzóska, M. M.; Galazyn-Sidorczuk, M.; Jurczuk, M.; Tomczyk, M. Protective effect of *Aronia melanocarpa* polyphenols on cadmium accumulation in the body: A study in a rat model of human exposure to this metal. *Curr. Drug Targets*, **2015**, *16*, 1470–1487. https://doi.org/10.2174/1389450116666150102121708
2. Dastan, D.; Karimi, S.; Larki–Harchegani, A.; Nili–Ahmadabadi, A. Protective effects of *Allium hirtifolium* Boiss extract on cadmium-induced renal failure in rats. *Environ. Sci. Pollut. Res.*, **2019**, *26*, 18886–18892. https://doi.org/10.1007/s11356–019–04656–7
3. Baiomy, A. A.; Mansour, A. A. Genetic and histopathological responses to cadmium toxicity in rabbit's kidney and liver: Protection by ginger (*Zingiber officinale*). *Biol. Trace Elem. Res.*, **2016**, *170*, 320–329. https://doi.org/10.1007/s12011-015-0491-4
4. Gabr, S. A.; Alghadir, A. H.; Ghoniem, G. A. Biological activities of ginger against cadmium-induced renal toxicity. *Saudi J. Biol. Sci.*, **2019**, *26*, 382–389. https://doi.org/10.1016/j.sjbs.2017.08.008
5. Suliman Al–Gebaly A. Ameliorative effect of *Arctium lappa* against cadmium genotoxicity and histopathology in kidney of Wistar rat. *Pak. J. Biol. Sci.*, **2017**, *20*, 314–319. https://doi.org/10.3923/pjbs.2017.314.319
6. Ramamurthy, C. H.; Subastri, A.; Suyavaran, A.; Subbaiah, K. C.; Valluru, L.; Thirunavukkarasu, C. *Solanum torvum* Swartz. fruit attenuates cadmium-induced liver and kidney damage through modulation of oxidative stress and glycosylation. *Environ. Sci. Pollut. Res.*, **2016**, *23*, 7919–7929. https://doi.org/10.1007/s11356-016-6044-3
7. Jung, S. Y.; Kim, S.; Lee, K.; Kim, J. Y.; Bae, W. K.; Lee, K.; Han, J.-S.; Kim, S. Association between secondhand smoke exposure and blood lead and cadmium concentration in community dwelling women: the fifth Korea National Health and Nutrition Examination Survey (2010–2012). *BMJ Open*, **2015**, *5*, e008218. https://doi.org/10.1136/bmjopen-2015-008218
8. El–Aziz, A. G. S.; Mustafa, H. N.; Saleh, H. A.; El–Fark M. M. O. *Zingiber Officinale* alleviates maternal and fetal hepatorenal toxicity induced by prenatal cadmium. *Biomed. Pharmacol. J.*, **2018**, *11*, 1369–1380. http://dx.doi.org/10.13005/bpj/1500
9. Gattea Al-Rikabi, Z.; Abbas, A. H.; Kadhum Oudah, H.; Sajer Nassir, H.; Ali, S. A. Histopathological study of liver and kidney tissues in C57 mice via chronic exposure to cadmium and zinc. *Arch. Razi Inst.*, **2021**, *76*, 1501–1508. https://doi.org/10.22092/ari.2021.355622.1705
10. Sanjeev, S.; Bidanchi, R. M.; Murthy, M. K.; Gurusubramanian, G.; Roy, V. K. Influence of ferulic acid consumption in ameliorating the cadmium-induced liver and renal oxidative damage in rats. *Environ. Sci. Pollut. Res.*, **2019**, *26*, 20631–20653. https://doi.org/10.1007/s11356–019–05420–7
11. Huang, K.; Deng, Y.; Yuan, W.; Geng, J.; Wang, G.; Zou, F. Phospholipase D1 ameliorates apoptosis in chronic renal toxicity caused by low-dose cadmium exposure. *Biomed Res. Int.*, **2020**, 7091053. https://doi.org/10.1155/2020/7091053
12. Elkhadragy, M. F.; Al–Olayan, E. M.; Al–Amiery, A. A.; Abdel Moneim, A. E. Protective effects of *Fragaria ananassa* extract against cadmium chloride-induced acute renal toxicity in rats. *Biol. Trace Elem. Res.*, **2018**, *181*, 378–387. https://doi.org/10.1007/s12011–017–1062–7
13. Poontawee, W.; Natakankitkul, S.; Wongmekiat, O. Protective effect of *Cleistocalyx nervosum var. paniala* fruit extract against oxidative renal damage caused by cadmium. *Molecules*, **2016**, *21*, 133. https://doi.org/10.3390/molecules21020133
14. Shen, R.; Liu, D.; Hou, C.; Liu, D.; Zhao, L.; Cheng, J.; Wang, D.; Bai, D. Protective effect of *Potentilla anserina* polysaccharide on cadmium-induced nephrotoxicity in vitro and in vivo. *Food Funct.*, **2017**, *8*, 3636–3646. https://doi.org/10.1039/c7fo00495h
15. Akinyemi, A. J.; Faboya, O. L.; Paul, A. A.; Olayide, I.; Faboya, O. A.; Oluwasola, T. A. Nephroprotective effect of essential oils from ginger (*Zingiber officinale*) and turmeric (*Curcuma longa*) rhizomes against cadmium-induced nephrotoxicity in rats. *J. Oleo Sci.*, **2018**, *67*, 1339–1345. https://doi.org/10.5650/jos.ess18115
16. Athmouni, K.; Belhaj, D.; Chawech, R.; Jarraya, R.; El Feki, A.; Ayadi, H. Characterization of polysaccharides isolated from *Periploca angustifolia* and its antioxidant activity and renoprotective potential against cadmium induced toxicity in HEK293 cells and rat kidney. *Int. J. Biol. Macromol.*, **2019**, *125*, 730–742. https://doi.org/10.1016/j.ijbiomac.2018.12.046
17. Ojo, O. A.; Ajiboye, B. O.; Oyinloye, B. E.; Ojo, A. B.; Olarewaju, O. I. Protective effect of *Irvingia gabonensis* stem bark extract on cadmium-induced nephrotoxicity in rats. *Interdiscip. Toxicol.*, **2014**, *7*, 208–214. https://doi.org/10.2478/intox-2014-0030
18. Wang, D.; Sun, H.; Wu, Y.; Zhou, Z.; Ding, Z.; Chen, X.; Xu, Y. Tubular and glomerular kidney effects in the Chinese general population with low environmental cadmium exposure. *Chemosphere*, **2016**, *147*, 3–8. https://doi.org/10.1016/j.chemosphere.2015.11.069
19. Weaver, V.; Navas-Acien, A.; Tellez-Plaza, M.; Guallar, E.; Muntner, P.; Silbergeld, E.; Jaar, B. Blood cadmium and lead and chronic kidney disease in US adults: A joint analysis. *Am. J. Epidemiol.*, **2009**, *170*, 1156–1164. https://doi.org/10.1093/aje/kwp248
20. Madrigal, J. M.; Ricardo, A. C.; Persky, V.; Turyk, M. Associations between blood cadmium concentration and kidney function in the U.S. population: Impact of sex, diabetes and hypertension. *Environ. Res.*, **2019**, *169*, 180–188. https://doi.org/10.1016/j.envres.2018.11.009
21. Ferraro, P. M.; Costanzi, S.; Naticchia, A.; Sturniolo, A.; Gambaro, G. Low level exposure to cadmium increases the risk of chronic kidney disease: Analysis of the NHANES 1999-2006. *BMC Public Health*, **2010**, *10*, 304. https://doi.org/10.1186/1471-2458-10-304
22. Lin, Y. S.; Ho, W. C.; Caffrey, J. L.; Sonawane, B. Low serum zinc is associated with elevated risk of cadmium nephrotoxicity. *Environ. Res.*, **2014**, *134*, 33–38. https://doi.org/10.1016/j.envres.2014.06.013
23. Jain, R. B. Co-exposures to toxic metals cadmium, lead, and mercury and their impact on unhealthy kidney function. *Environ. Sci. Pollut. Res.*, **2019**, *26*, 30112–30118. https://doi.org/10.1007/s11356-019-06182-y

24. Grau-Perez, M.; Pichler, G.; Galan-Chilet, I.; Briongos-Figuero, L. S.; Rentero-Garrido, P.; Lopez-Izquierdo, R.; Navas-Acien, A.; Weaver, V.; García-Barrera, T.; Gomez-Ariza, J. L.; et al. Urine cadmium levels and albuminuria in a general population from Spain: A gene-environment interaction analysis. *Environ. Int.*, **2017**, *106*, 27–36. https://doi.org/10.1016/j.envint.2017.05.008
25. Kim, N. H.; Hyun, Y. Y.; Lee, K. B.; Chang, Y.; Rhu, S.; Oh, K. H.; Ahn, C. Environmental heavy metal exposure and chronic kidney disease in the general population. *J. Korean Med. Sci.*, **2015**, *30*, 272–277. https://doi.org/10.3346/jkms.2015.30.3.272
26. Wu, C. Y.; Wong, C. S.; Chung, C. J.; Wu, M. Y.; Huang, Y. L.; Ao, P. L.; Lin, Y. F.; Lin, Y. C.; Shiue, H. S.; Su, C. T; et al. The association between plasma selenium and chronic kidney disease related to lead, cadmium and arsenic exposure in a Taiwanese population. *J. Hazard. Mater.*, **2019**, *375*, 224–232. https://doi.org/10.1016/j.jhazmat.2019.04.082
27. Eom, S. Y.; Seo, M. N.; Lee, Y. S.; Park, K. S.; Hong, Y. S.; Sohn, S. J.; Kim, Y. D.; Choi, B. S.; Lim, J. A.; Kwon, H. J; et al. Low-level environmental cadmium exposure induces kidney tubule damage in the general population of Korean adults. *Arch. Environ. Contam. Toxicol.*, **2017**, *73*, 401–409. https://doi.org/10.1007/s00244-017-0443-4
